# Equivalence of Type 2 Diabetes Prevalence Estimates: Comparative Study of Similar Phenotyping Algorithms Using Electronic Health Record Data

**DOI:** 10.2196/79653

**Published:** 2025-10-27

**Authors:** Muchiri E Wandai, Katie S Allen, Ashley Wiensch, John Price, Brian E Dixon

**Affiliations:** 1 Regenstrief Institute, 1101 West 10th Street, Indianapolis, IN, 46202, United States, 1 317-274-9000; 2 Richard M. Fairbanks School of Public Health (FSPH), Indiana University, Indianapolis, IN, United States

**Keywords:** type 2 diabetes, computable phenotype, prevalence, equivalence, two one-sided tests, TOST, electronic health record, EHR, public health surveillance, public health research, public health practice, health information exchange

## Abstract

**Background:**

Timely surveillance of diabetes mellitus remains a challenge for public health agencies. In this study, researchers compared type 2 diabetes (T2D) prevalence estimates using electronic health record (EHR) data and computable phenotypes (CPs) as defined and applied by 2 independent networks. One network, Diabetes in Children, Adolescents, and Young Adults, was a research consortium, and the other, the Multi-State EHR-Based Network for Disease Surveillance, is a practice-based public health surveillance network.

**Objective:**

This study sought to determine the equivalence of T2D prevalence estimates generated by 2 distinct, yet conceptually related, CPs using EHR data.

**Methods:**

Each network used diagnostic, laboratory, and medication data for young adults (aged 18-44 years) extracted from the Indiana Network for Patient Care (INPC) to independently calculate prevalence of T2D using distinct CPs for the year 2022. The INPC is a statewide health information exchange that receives EHR data from multiple health care systems and supports public health use cases such as surveillance. The two one-sided tests method for independence with a predefined margin of –2.5 to +2.5 percentage points was used to compare the estimated prevalence as previously derived from the Multi-State EHR-Based Network for Disease Surveillance and Diabetes in Children, Adolescents, and Young Adults networks. The two one-sided tests for equivalence show that any observed difference between 2 estimates is small and practically insignificant. Results at the overall level, and stratified by sex, age, and race or ethnicity, were examined.

**Results:**

Overall prevalence estimates for 2022 were 4.1% for CP 1 and 2.4% for CP 2. Although prevalence estimates for CP 1 were consistently higher than those for CP 2, absolute differences were generally less than 2.5 percentage points, which did not result in a statistically significant (*P*<.001) difference between estimates. The only exception was for Hispanic individuals, where prevalence was significantly different (*P*=0.2) for CP 1 (5.4%) versus CP 2 (3.0%), yielding a margin of 2.4 (95% CI 2.2-2.6) percentage points. Other groups that had relatively higher but statistically nonsignificant prevalence included male individuals (4.6% for CP 1 vs 2.3% for CP 2), individuals aged 35-44 years (6.9% for CP 1 vs 4.9% for CP 2), and African American individuals (5.5% for CP 1 vs 3.7% for CP 2). Therefore, we concluded that the 2 CPs largely produced equivalent estimates of T2D prevalence.

**Conclusions:**

The 2 independent CPs demonstrated equivalent T2D prevalence estimates, except in Hispanic individuals. Although the CPs can be considered statistically equivalent, the data driving each CP may impact accuracy and completeness. CP 1 was broader, incorporating clinical diagnoses, laboratory data, and medication, whereas CP 2 used clinical diagnostic codes alone. These results have implications for improving harmonization of CPs for public health surveillance.

## Introduction

### Background

Diabetes is a major public health concern as it poses numerous health challenges that increase the risk of complications such as heart disease, kidney failure, and vision loss [1]. This chronic disease also has a significant impact on individuals’ quality of life [2,3], health care systems, and economies, particularly when left untreated or poorly managed [4]. Timely interventions to prevent or delay the onset of diabetes are a common, critical function performed by public health organizations.

In the United States, there has been a rising prevalence of diabetes over the past few decades [5]. Between August 2021 and August 2023, the prevalence of diabetes among the adult population (aged ≥20 years) was 15.8%, which included both diagnosed (11.3%) and undiagnosed (4.5%) cases [6], with type 2 diabetes (T2D) accounting for approximately 90% to 95% of these instances [7]. The prevalence of diabetes varies by sex, age, race, and ethnicity, with higher rates observed among certain groups, including African American, Hispanic, and American Indian or Alaska Native individuals [8]. Risk factors contributing to diabetes include obesity, physical inactivity, and a family history of the disease [9].

In the US state of Indiana, the prevalence of diabetes mirrors national trends. According to recent data (2023) from the Behavioral Risk Factor Surveillance System, 13.2% of Indiana adults (aged ≥18 years) have been diagnosed with diabetes, with T2D being the most common [10]. The state exhibits notable disparities in diabetes prevalence based on factors such as age, race, and socioeconomic status. Higher rates are often observed among African American and Hispanic populations, as well as in individuals with lower incomes and educational levels [11]. Estimating prevalence at the state and substate levels is challenging due to the small sample sizes in existing surveillance systems. The National Health and Nutrition Examination Survey and the National Health Interview Survey that monitor the prevalence and incidence of diabetes in the United States have been shown to fall short of large enough sample sizes to accurately assess diabetes prevalence and incidence in youth at the state or national levels, as well as in race and ethnicity subgroups [12]. In addition, differentiating diagnosis by diabetes type in children, adolescents, and young adults has also been challenging due to the co-occurrence between T2D and obesity [13]. Therefore, minor variations in the way in which a case is defined for these age groups could yield huge differences in prevalence, unlike that in the middle-aged and older adults.

Given the limitations of existing surveillance systems, there is an increasing interest in using electronic health record (EHR) data to estimate prevalence at these granular levels. It is posited that EHR systems may capture more cases with distinguishing characteristics relative to a population-based survey as data are obtained from all individuals interacting with health care services [14]. Accurate estimates of the burden of diseases are critical in policy advising, planning of prevention and control programs, and optimal allocation of scarce resources [15-17]. Specifically, accurately estimating the burden of diabetes in unique geographic areas is essential for improving overall health outcomes and reducing health care costs [11].

One aspect of accurately estimating the burden is ensuring precision in estimating the prevalence of individuals with diabetes. This is particularly true when leveraging EHR data, which may be derived from various sources such as hospital records and administrative data. Identifying accurate case definitions is a critical component in estimating prevalence using EHR data [18]. It has been shown that the prevalence of diabetes can vary based on differences in computable phenotype (CP) definitions, which are sets of clinical features used to identify a patient with a particular condition [19]. The variation is dependent on component criteria and timing of observations and measurements, and as such, phenotype definition requires careful consideration if EHR data are to be useful for individual and population health improvement [20].

### Objectives

Understanding case definitions for a particular health condition is important, as this would determine the degree to which disease surveillance measures could be under-, over-, or accurately estimated. EHRs contain a variety of data, including diagnoses, laboratory test results, and medications. The methods through which these data are combined in a CP could affect the accuracy of measures such as incidence and prevalence. The purpose of this study was to compare 2 CP methods for estimating the prevalence of diabetes using EHR data. These methods have been proposed for use by researchers as well as local and state public health agencies. The findings could be useful for public health practitioners in diabetes prevention and control programs to identify the method that best captures their populations and to understand the strengths and limitations of each approach.

## Methods

### Study Overview

The primary objective of this study was to compare T2D prevalence rates calculated from EHR data during the same period (2022) for young adults living in the Indianapolis metropolitan area using 2 previously defined CPs. The rates were calculated using Indiana-based data included in 2 national networks that seek to develop generalizable methods for using EHR data to enhance surveillance of chronic disease in the United States.

The first CP (CP 1) is based on the Multi-State EHR-Based Network for Disease Surveillance (MENDS) led by the National Association of Chronic Disease Directors, with 5 distinct geographic sites [21]. The second CP (CP 2) was developed by the Diabetes in Children, Adolescents, and Young Adults (DiCAYA) network (coordinated by New York University Langone), which has 8 distinct geographic sites [22].

Both projects are funded by the US Centers for Disease Control and Prevention and have been previously described in the literature. Indiana is a common site in both the networks. Through secondary analysis, we sought to understand whether the 2 sets of estimates converged or diverged from one another and examine the potential reasons for convergence or divergence. The two one-sided tests (TOST) method was used to find a minimum, non-nil effect size between the prevalences produced by the 2 algorithms. Expert knowledge based on the available literature was used to determine whether the minimum effect size obtained can be used to infer practically equivalent prevalences.

### Data Sources

The data used in each source study were extracted from the Indiana Network for Patient Care (INPC), the EHR repository at the center of the Indiana Health Information Exchange. The Indiana Health Information Exchange is a network of >20 years, including >100 hospitals, clinical laboratories, outpatient clinics, and other health care organizations that exchange EHR data for clinical and population health purposes [23,24]. The INPC is routinely used to support research [25]. The INPC hosts a variety of EHR data, both structured and unstructured, including diagnosis codes; laboratory tests; clinical observation reports in text form; and vital measurements such as blood pressure, body weight, and height. Data on diabetes diagnosis, treatment, and laboratory measurements previously extracted by the 2 research consortiums were used in this study.

Data from the INPC were extracted independently by each network study to calculate their respective CPs. These data were subsequently available to the researchers to use in this comparative study. The time windows for observation and prevalence measurement overlapped in the year 2022. Therefore, we used January 1, 2022, to December 31, 2022, as the prevalence window of observation.

### Populations

CP 1 used a subset of the INPC data, focusing on individuals who sought care from 2 health systems that collectively deliver care to at least two-thirds of the population in central Indiana. One system, the largest in the state, has 17 hospitals, 15 emergency rooms, 11 urgent care centers, nearly 3000 hospital beds, and hundreds of primary care and specialty clinics. The second system, based in the city of Indianapolis, has over 300 beds, an emergency room, outpatient and inpatient care, specialty clinics, and 12 community health centers. Combined, the 2 health systems delivered care to 2.5 million individuals during the observation window. CP 1 included patients with at least one clinical encounter during a 2-year period, including the year of observation for diabetes prevalence, to capture a representative number of clinical encounters since individual health care use may not take place annually, especially for individuals who consider themselves to be healthy.

CP 2 used a subset of the INPC data that included individuals aged 18 to 44 years living in the metropolitan statistical area (MSA) of Indianapolis. The project included data from all health care providers during the observation window to patients living in the 11 counties that constitute the MSA. The total population in the MSA is 2.7 million. Included patients were required to have had at least one clinical encounter in the previous 3 years, which included the year of prevalence observation and the previous 2 years.

Because the DiCAYA study that developed CP 2 limited its scope to patients aged 18 to 44 years, we compared prevalence measures for only that age range. This allowed the study to be conducted without reidentifying patients or pulling additional data from the INPC. Furthermore, ,the distribution of the INPC encounter data (used as the denominator) by sex, race or ethnicity, and age was compared with that of the American Community Survey. This helped in understanding whether any group was nonrepresentative of the underlying population.

### CP Description

Textbox 1 provides a summary of each CP algorithm, allowing for a comparison of the distinct approaches used in each surveillance project. We kept the geographic and CP distinctions intact.

Textbox 1. **Comparison of the type 2 diabetes (T2D) computable phenotype (CP) algorithms examined in this study.**

**CP 1 from Multi-State Electronic Health Record–Based Network for Disease Surveillance [** **26** **]**

Starts by diabetes defined as: Any one of the following:

1. Laboratory:
  - Hemoglobin A_1C_ ≥6.5
  - Fasting glucose ≥126
  - Random glucoses ≥200 on 2 or more occasions within a 2-year period
2. Diagnostic codes: *International Classification of Diseases* (*ICD*) codes on 2 or more occasions within a 2-year period
  - *ICD-9* code 250.x or
  - *ICD-10* code (E10.x, E11.x, or E14.x)
3. Prescriptions:Prescription for INSULIN outside of pregnancyOther prescriptions: albiglutide, alogliptin, chlorpropamide, dulaglutide, ertugliflozin, exenatide, glimepiride, glipizide, glyburide, liraglutide, lixisenatide, pramlintide, rosiglitazone, semaglutide, sitagliptin, tolazamide, tolbutamide, acarbose, canagliflozin, dapagliflozin, empagliflozin, gliclazide, linagliptin, miglitol, nateglinide, pioglitazone, repaglinide, saxagliptin, tirzepatide

Type 1

Among patients meeting the definition for diabetes above, define as type 1 diabetes (T1D) if any of the following apply:

1. C-peptide test <0.8
2. Diabetes auto-antibodies positive
3. Prescription for URINE ACETONE TEST STRIPS
4. Ratio of T1D: T2D *ICD-9* or *ICD-10* codes >50% and prescription for GLUCAGON
5. Ratio of T1D: T2D *ICD-9* or *ICD-10* codes >50% and never prescribed oral hyperglycemic medications

Type 2: if not T1D

**CP 2 from Diabetes in Children, Adolescents, and Young Adults [** **27** **]**

Step 1: Starts by a wide-net definition of potential patients with diabetes mellitus (DM) meeting any of the following:

1. ≥1 Hemoglobin A1c ≥6.5% (≥42 mmol/mol)
2. ≥1 Fasting glucose ≥126 mg/dL (≥7.0 mmol/L)
3. ≥1 Random plasma glucose ≥200 mg/dL (≥11.1 mmol/L)
4. ≥1 Diabetes-related diagnosis code from an inpatient or outpatient encounter
5. ≥1 Prescribed, administered (ie, provided during a hospitalization), or dispensed diabetes-related medication

Medications: ≥1 prescribed, dispensed, or administered: exenatide, gliclazide, glimepiride, glipizide, glyburide, insulin, meglitinide, metformin, nateglinide, pioglitazone, pramlintide, repaglinide, rosiglitazone, sitagliptin, sulfonylurea, thiazolidinediones.

Step 2: Uses the pool of patients in Step 1 to identify DM cases using diagnostic codes only.*ICD-9* codes: 250.x0 and 250.x2*ICD-10* codes: E08.x, E10.x, E11.x, E13.x

Prevalent T2D:

1. $\frac{\#\text{ type 1 diagnosis codes}}{\#\text{ DM diagnosis codes}}\leq 0.5$; OR
2. Evidence of an anti-diabetes medication besides insulin or metformin; OR
3. No evidence of glucagon medication

CP 1 distinguishes diabetes by type (type 1 diabetes [T1D] or T2D) based on laboratory measurements (glycated hemoglobin [HbA_1c_]≥6.5%, fasting plasma glucose ≥126 mg/dL, and random plasma glucose ≥200 mg/dL) on 2 or more occasions within a 2-year period; C-peptide and diabetes auto-antibody tests; *International Classification of Diseases, Tenth Revision* (*ICD-10*), code (E10.x, E11.x, or E14.x) on 2 or more occasions within a 2-year period; and prescriptions of insulin outside of pregnancy, oral hyperglycemic medications, or urine acetone test strips. Patients are classified as having T1D if any of the following criteria are met: C-peptide test of <0.8 ng/mL, positive diabetes auto-antibody test, prescription for urine acetone test strips, ratio of T1D to T2D *ICD-10* codes of >50%, and prescription for glucagon and never having been prescribed oral hypoglycemic medications. A patient who does not meet any of the aforementioned criteria for T1D is classified as having T2D.

CP 2 uses a 2-step process to determine whether a patient has T2D. First, data on diagnoses (*ICD-10, Clinical Modification,* codes E08.x, E09.x, E10.x, E11.x, and E13.x); laboratory test results (HbA_1c_>6.5% and fasting glucose≥126 mg/dL); or medications (eg, metformin) for patients with at least 2 clinical encounters are extracted. Next, the CP classifies individuals by diabetes type based only on the *ICD-10* diagnosis codes. A patient is classified as having T2D if the ratio of the number of T1D codes to the total number of diabetes codes per patient is less than half (<0.50) [22].

Textbox 1 shows that the main difference between the CPs is that CP 2 does not include medication and laboratory data, whereas CP 1 excludes the E08, E09, and E13 *ICD-10* diagnostic codes. Diagnostic codes E10.x and E11.x are included in both CPs.

### Statistical Analysis

The TOST method of equivalence for independent samples was used to test whether diabetes prevalence differed by a prespecified margin of equivalence based on the definition of T2D by the different CPs from MENDS and DiCAYA. The TOST procedure is used to statistically reject the presence of effects that are large enough to be considered worthwhile [28]. This method shows whether the difference between groups is smaller than a tolerably small amount [29]. T2D prevalence was stratified by sex, age group, and race. The choice of the equivalence interval for statistical and practical significance is critical [29,30]. In this study, we used a margin of 2.5 percentage points as recommended by Tatem et al [31] for estimates that are below 10%. A significance level (α) of .05 was used, yielding 90% CIs for equivalence testing because the formula for the CI in the TOST method is 100(1 – 2α)% [32]. We hypothesized that prevalence estimates based on CP 1 and CP 2 would differ by more than 2.5 percentage points (as recommended by Tatem et al [31]); that is, the difference in the prevalences would lie outside the −2.5 to +2.5 bounds.

The comparison involved the difference in prevalence calculated as a result of the independent CP methods used in the distinct studies. Therefore, a TOST of independence was used given that each network study pulled data and calculated prevalence independently. Although the population in the MENDS study accounts for 80% of the geographic region covered by the DiCAYA study, which implies that all MENDS patients would likely be included in the DiCAYA study, the researchers could not link individual patient records used in the 2 network studies. Therefore, we treated the datasets from each network study as independent.

### Ethical Considerations

#### Ethics Review and Approval

This study was conducted under an approved research protocol by the Human Research Protection Program at Indiana University (protocol 2008160551), which received exemption under Category 4(iii) of the revised Common Rule.

#### Privacy and Informed Consent

This study represents a secondary analysis of deidentified, population-level EHR data obtained from the INPC, which is governed by HIPAA (Health Insurance Portability and Accountability Act) protections as it is a health information exchange (HIE) that uses and discloses protected health information. The data used in this research contained no personally identifiable information as all data were anonymized, and the researchers did not have access to any patient identifiers. Because no human participants were directly involved in the research, the institutional review board waived the need for researchers to obtain informed consent.

## Results

The overall prevalence of T2D among young adults in the year 2022 was 4.1% for CP 1 and 2.4% for CP 2 (Table 1). A breakdown by the demographic variables (age, sex, and race) also showed that CP 1–based prevalences were higher than those estimated using CP 2. However, despite numeric differences in the prevalence estimates, the variation was not more than 2 percentage points, with 3 notable exceptions: individuals aged 35 to 44 years, Hispanic individuals, and male individuals. Moreover, the only group for which the null hypothesis was not rejected was Hispanic individuals (difference of 2.4%, 95% CI 2.2%-2.6%). Both CPs produced narrow CIs under the assumption of normally distributed prevalences. Therefore, despite the differences, both CPs on average showed statistically equivalent estimates of diabetes prevalence for the Indianapolis MSA. However, it is important to note that the difference in prevalence between the 2 CPs increased with age. Therefore, there is a likelihood that the CPs could show no equivalent estimates for older ages.

**Table 1.** Estimated prevalence of type 2 diabetes derived from 2 different computable phenotypes (CPs) for the Indianapolis metropolitan statistical area population by age, sex, and race (2022).

Demographic characteristic	CP 1, n/N (%)	CP 2, n/N (%)	Percentage point difference (90% CI)	Reject null hypothesis?
Age group (y)				
18-24	1506/88,952 (1.7)	412/196,113 (0.2)	1.5 (1.4 to 1.6)	Yes
25-34	4105/128,848 (3.2)	4568/302,385 (1.5)	1.7 (1.6 to 1.8)	Yes
35-44	8394/121,714 (6.9)	13,589/276,832 (4.9)	2 (1.9 to 2.1)	Yes
Race				
African American	3401/62,064 (5.5)	4393/118,634 (3.7)	1.8 (1.6 to 2)	Yes
Asian	353/8900 (4.0)	696/28,771 (2.4)	1.6 (1.2 to 2)	Yes
Hispanic	2063/38,432 (5.4)	2253/74,144 (3.0)	2.4 (2.2 to 2.6)	No
White	7853/210,135 (3.7)	9706/457,682 (2.1)	1.6 (1.5 to 1.7)	Yes
Other	335/19,983 (1.7)	1521/96,099 (1.6)	0.1 (–0.1 to 0.3)	Yes
Sex				
Female	8009/207,979 (3.9)	10,488/428,534 (2.4)	1.5 (1.4 to 1.6)	Yes
Male	5996/131,535 (4.6)	8081/346,796 (2.3)	2.3 (2.2 to 2.4)	Yes
Overall	14,005/339,514 (4.1)	18,569/775,330 (2.4)	1.7 (1.6 to 1.8)	Yes

## Discussion

### Principal Findings

This study compared prevalence estimates of T2D for a single observation period using EHR data among young adults from 2 networks (MENDS and DiCAYA) that use distinct CP algorithms. The 2 CPs were developed independently to achieve generalizable methods for surveillance of diabetes mellitus (DM) by type using EHR data. CP 1 was developed in concert with local health departments and was designed to be used at the local and state levels. This CP incorporates diagnostic codes, laboratory measures, and medications to classify patients with T2D. CP 2 was developed by diabetes and epidemiological researchers to be used for obtaining state and national estimates of prevalence. This CP uses only diagnostic codes in its determination of T2D classification. On the basis of the results, the CPs produced equivalent estimates of the prevalence of T2D (except among Hispanic individuals) despite differences in their individual point estimates and in their approaches.

Our methodological approach to equivalence testing and interpretation of the findings is supported by prior work using the TOST method. Using a difference of –2.5 to +2.5 percentage points between prevalence estimates to indicate equivalence is reasonable and in line with prior work that tested various margins. For example, a study comparing equivalence of prevalence estimates from population survey data found a difference of –5 to +5 percentage points useful in establishing equivalence but a lower margin of –2.5 to +2.5 percentage points in cases in which prevalence was below 10% [31]. A margin of –5 to +5 percentage points was also used in a study investigating disparity in pediatric immunization coverage by race and ethnicity [33]. Given the variation in ranges, we based our decision on the recommendations of Tatem et al [31] for estimates that are below 10%.

The findings of this study have implications for EHR-based CP development and refinement, as well as for their (CPs) use in research. These findings also have implications for public health practice, which seeks to use CPs off the shelf for the surveillance of diabetes and other chronic diseases.

### Considerations for CP Algorithms

Although the CPs were equivalent, they are fundamentally different, and these differences have implications for the accuracy of the prevalence estimates. On average, CP 1 produced higher estimates of T2D prevalence than CP 2. There are several reasons for these differences. First, although the CP 2 algorithm initially considers a range of clinical variables (eg, fasting glucose, HbA_1c_, and medications) in determining the population of patients likely to have diabetes, at the classification stage of the algorithm, it only uses *ICD-10* codes to distinguish between T1D and T2D. On the other hand, CP 1 includes individuals in the numerator based on the diagnostic codes, presence of 1 or more abnormal laboratory measurements, and medications. Second, the CP 1 and CP 2 DM diagnostic codes differ slightly. The CP 1 algorithm does not use the E08.x, E09.x, and E13.x codes but does include E14.x, all of which represent other or unidentified DM types. On the basis of CP 1, it is likely that patients with E08.x, E09.x, or E13.x who have an abnormal laboratory test result will be classified as having T2D and not other DM types. This could be a factor contributing to Hispanic individuals showing a nonequivalent prevalence between the CPs, especially if they (Hispanics) were more sensitive to the discrepant items defining prevalence between the CPs (they could be having a slightly higher number of patients that are flagged as having diabetes based on laboratory tests and on *ICD-10* diagnostic codes E08.x, E13.x, or E14.x). This difference might not be important except when considering accuracy, as E09.x might be temporary due to the influence of other drugs rather than an indication of chronic illness.

As work continues to improve the accuracy, sensitivity, and specificity for diabetes CPs and other chronic diseases, these differences should be noted and further analyzed. Our results demonstrate not just equivalence but the need for refinement and harmonization. Using a broader set of diagnostic criteria, such as the laboratory data used by CP 1, likely improves accuracy and sensitivity as prior work has found that using diagnostic codes alone in EHR data can generate inaccurate estimates of prevalence [34,35]. Moreover, the value of medication data should be reconsidered in light of prior work as some diabetes medications are prescribed for nondiabetes conditions such as obesity and heart failure [36].

### Considerations for Public Health Practice

Public health indicators, such as the incidence and prevalence of chronic diseases, provide insights into the health status of a population, allowing public health officials to monitor trends, identify areas of concern, evaluate the effectiveness of interventions, and inform policy decisions by highlighting key health issues and areas requiring further action [37-39]. The ultimate objective in calculating health indicators is to improve the health of populations and reduce preventable inequalities [40]. These indicators are estimates of a given health dimension in a target population and are therefore prone to some degree of imprecision [41]. Thus, it is essential to consider a test of equivalence of these indicators when measuring from different data sources or when using different methodological algorithms.

This study found that diabetes prevalence estimated from 2 slightly divergent phenotype algorithms can be considered equivalent, except when measuring prevalence in Hispanic populations. This implies that some jurisdictions, which might only have *ICD-10* codes available or data from a limited number of health systems in their geographic area, could estimate prevalence in a reasonably equivalent manner to those jurisdictions with access to near complete coverage or with full EHR data available. This could be an option for some public health organizations as prevalence computation may be quicker. Jurisdictions may now be able to implement routine estimates for chronic illness with limited data, improving timeliness while they continue efforts to implement data modernization plans. In addition, leaner approaches to population estimates may be necessary if funding for chronic disease or surveillance programs is reduced in the following months or years.

We note that although the CPs are implementable, they are complex. The sites involved in both the MENDS and DiCAYA studies are sophisticated health systems, academic medical centers, or HIE networks. Public health does not yet have scalable, easy-to-implement applications that analyze *ICD-10* data for chronic disease surveillance. Moreover, no library of CPs for chronic disease surveillance exists that could be easily downloaded and implemented within jurisdictions’ computing environments. More work is needed to make CP algorithms available to public health jurisdictions for implementation, along with tools that support knowledge management and integration into the existing surveillance infrastructure. Future work should focus on improving not only the accuracy of CP algorithms but also the distribution, implementation, and maintenance of such algorithms for use by thousands of public health agencies. Although platforms such as the National Coalition of Certification Centers (NC3) knowledge base and the eMERGE network exist for clinical research studies, these efforts may not meet the needs of public health agencies. Therefore, public health jurisdictions may need to create a separate platform for sharing CP algorithms or partner with efforts such as NC3 and eMERGE to distinguish clinical CPs from those designed for public health use.

### Limitations

This study has several limitations of note. First, the denominators for each CP were not the same. CP 2 leveraged the entire population of the Indianapolis MSA who sought care during the observation window, whereas CP 1 used only patients from 2 health systems. This limitation is mitigated by the fact that the 2 health systems, data from which were used by both CPs, represent more than two-thirds of the metropolitan population. However, it remains a potential source of bias for the CP 1–based estimates, which could be responsible for the significant difference in the Hispanic population between the 2 CPs (the numerators were comparable, but the CP 2 denominator was twice that of CP 1).

Moreover, it was not possible to link individual patient records between the MENDS and DiCAYA study data to independently verify whether the same patients with T2D were correctly identified by each CP algorithm as this was a secondary analysis using limited datasets generated for each independent network study. Applying the CP algorithms on the same set of patients and examining reasons for differences in classifications would strengthen reliability in the TOST findings, and this approach is recommended for future studies that compare CPs.

Additionally, medication data from all sources were limited. Medication data, including orders and retail pharmacy dispensing data, are not routinely available in the HIE network that provided the EHR data for both CPs. More complete medication data might improve the accuracy of the CPs, although prior work has found that few patients only have documentation of medication use without a chronic illness diagnosis [34]. However, relying on medication data to identify T2D cases may introduce misclassification bias as several antidiabetic medications such as metformin, glucagon-like peptide–1 receptor agonists, and sodium/glucose cotransporter 2 inhibitors are also prescribed for nondiabetic conditions, including polycystic ovary syndrome, obesity, and heart failure. This overlap can result in the inclusion of individuals without T2D, potentially inflating prevalence estimates and affecting phenotype specificity.

Finally, it was not possible to conduct extrinsic validation of the results using external datasets such as those from the Behavioral Risk Factor Surveillance System, claims data, or the US Diabetes Surveillance System (DSS). The 2022 data from the DSS showed that type-insensitive DM prevalence for the entire state of Indiana was 3.4% (95% CI 2.7%-4.3%) for individuals aged 18 to 44 years. However, it was not possible to obtain data from DSS stratified by sex, race or ethnicity, and granular age groups to compare with data from the MENDS or DiCAYA network studies. Comparisons with other public health data, such as in the study by Allen et al [42], are recommended in future research to assess external validity.

### Conclusions

As work continues to expand the use of EHR data for routine public health surveillance, such as the active work by jurisdictions within the Centers for Disease Control and Prevention data modernization initiative, refining CP algorithms for chronic diseases will be necessary to achieve the right balance between sensitivity and specificity. Efforts by both MENDS and DiCAYA demonstrate useful approaches that, through harmonization and refinement, can create practical and effective metrics that enable the tracking of diabetes burden over time. Future work is needed to standardize CP processes to enable scaling across the public health enterprise. This will enable not only surveillance but also action to reduce burden and improve health in communities.

## Abbreviations

CP: computable phenotype
DiCAYA: Diabetes in Children, Adolescents, and Young Adults
DM: diabetes mellitus
DSS: Diabetes Surveillance System
EHR: electronic health record
HbA_1c_: glycated hemoglobin
HIE: health information exchange
HIPAA: Health Insurance Portability and Accountability Act
*ICD-10*: *International Classification of Diseases, Tenth Revision*
INPC: Indiana Network for Patient Care
MENDS: Multi-State Electronic Health Record–Based Network for Disease Surveillance
MSA: metropolitan statistical area
T1D: type 1 diabetes
T2D: type 2 diabetes
TOST: two one-sided tests
